# Changes in the Daily Rhythm of Lipid Metabolism in the Diabetic Retina

**DOI:** 10.1371/journal.pone.0095028

**Published:** 2014-04-15

**Authors:** Qi Wang, Maria Tikhonenko, Svetlana N. Bozack, Todd A. Lydic, Lily Yan, Nicholas L. Panchy, Kelly M. Mcsorley, Matthew S. Faber, Yuanqing Yan, Michael E. Boulton, Maria B. Grant, Julia V. Busik

**Affiliations:** 1 Department of Physiology, Michigan State University, East Lansing, Michigan, United States of America; 2 Department of Microbiology and Molecular Genetics, Michigan State University, East Lansing, Michigan, United States of America; 3 Department of Chemistry, Michigan State University, East Lansing, Michigan, United States of America; 4 Department of Psychology Social Science, Michigan State University, East Lansing, Michigan, United States of America; 5 Department of Pharmacology and Therapeutics, University of Florida, Gainesville, Florida, United States of America; 6 Department of Ophthalmology, Indiana University, Indianapolis, Indiana, United States of America; Morehouse School of Medicine, United States of America

## Abstract

Disruption of circadian regulation was recently shown to cause diabetes and metabolic disease. We have previously demonstrated that retinal lipid metabolism contributed to the development of diabetic retinopathy. The goal of this study was to determine the effect of diabetes on circadian regulation of clock genes and lipid metabolism genes in the retina and retinal endothelial cells (REC). Diabetes had a pronounced inhibitory effect on the negative clock arm with lower amplitude of the *period* (*per*) *1* in the retina; lower amplitude and a phase shift of *per2* in the liver; and a loss of *cryptochrome* (*cry*) *2* rhythmic pattern in suprachiasmatic nucleus (SCN). The positive clock arm was increased by diabetes with higher amplitude of *circadian locomotor output cycles kaput* (*CLOCK*) and *brain and muscle aryl-hydrocarbon receptor nuclear translocator-like 1* (*bmal1*) and phase shift in *bmal1* rhythmic oscillations in the retina; and higher *bmal1* amplitude in the SCN. *Peroxisome proliferator-activated receptor* (*PPAR*) *α* exhibited rhythmic oscillation in retina and liver; *PPARγ* had lower amplitude in diabetic liver; *sterol regulatory element-binding protein* (*srebp*) *1c* had higher amplitude in the retina but lower in the liver in STZ- induced diabetic animals. Both of Elongase (*Elovl*) *2* and *Elovl4* had a rhythmic oscillation pattern in the control retina. Diabetic retinas lost *Elovl4* rhythmic oscillation and had lower amplitude of *Elovl2* oscillations. In line with the in vivo data, circadian expression levels of *CLOCK*, *bmal1* and *srebp1c* had higher amplitude in rat REC (rREC) isolated from diabetic rats compared with control rats, while *PPARγ* and *Elovl2* had lower amplitude in diabetic rREC. In conclusion, diabetes causes dysregulation of circadian expression of clock genes and the genes controlling lipid metabolism in the retina with potential implications for the development of diabetic retinopathy.

## Introduction

The circadian clock is comprised of transcriptional/translational feedback loops of clock genes. Clock genes, in turn, regulate hormonal secretion and metabolism in accordance with the environmental light–dark cycle through direct effects on a myriad of clock-controlled genes such as peroxisome proliferator-activated receptor (*PPARs*) [1]. In the mammalian circadian clock system, SCN is a “master” clock, with a series of “peripheral” clocks that are located in almost all the peripheral tissues [2]. The physiological rhythmicity in peripheral tissues is mainly controlled by local molecular clock with the SCN functioning as a synchronizer of peripheral oscillators [3]. Although light is the main synchronizer (Zeitgeber) of central circadian rhythmicity, various signals associated with food intake (or fasting) are potent synchronizers for secondary clocks in peripheral organs such as liver, which plays critical role in the development of metabolic syndrome/type 2 diabetes [4]. Approximately 10% of the genes exhibit circadian expression in the liver, indicating that circadian rhythmicity is important for hepatic physiology [5], [6].

It is well known that mammalian retina contains an independent circadian clock [7]. A few studies have demonstrated that the retinal circadian clock regulates many retinal functions such as retinal gene transcription, visual processing and photoreceptor viability [8], [9], [10]. Because retina is the only source of photic input to the SCN and peripheral tissues of the body, it is suggested that the interaction between retinal clock and SCN clock plays a key role in the circadian organization of entire organism [11]. Dysregulation of retinal circadian clock system was shown to contribute to an increased risk for the development of glaucoma [12].

Recent studies clearly demonstrated a link between circadian clock and metabolism. In murine models, disruption of key clock–related genes leads to metabolic abnormalities such as hyperphagia, obesity, and hyperleptinemia [13], [14]. In humans, change of internal circadian time (e.g., during shift work) is related to the development of obesity, type 2 diabetes, and cardiometabolic diseases [15]. Polymorphism of human circadian clock genes is associated with metabolic dysfunction [16], [17], [18]. Although circadian nature of the retina is well accepted, the role of circadian regulation of retinal metabolism has not been studied. Key transcriptional factors involved in fatty acid and cholesterol metabolism, PPARs and SREBP, are known to be under circadian control. They are expressed in the retina and dysregulation of circadian pattern of these factors in diabetes could lead to retinal metabolic abnormalities contributing to the development of diabetic retinopathy. Our previous study demonstrated that decreased expression levels of retinal fatty acid Elongases Elovl2, Elovl4 and Elovl6 play an important role in the pathogenesis of diabetic retinopathy [19]. The mechanism responsible for the down-regulation of Elovl2 and Elovl4 remains unclear, but circadian dysregulation of lipid metabolism through PPARα and SREBP-mediated pathways may be involved.

The purpose of this study was twofold: to first determine the circadian expression of clock and metabolic genes in the SCN, retina and liver and second to examine the effect of diabetes on clock genes, fatty acid metabolic pathways, and key transcription factors controlling fatty acid metabolism: SREBP 1C, PPARα and PPARγ.

## Materials and Methods

### Animals and Induction of Diabetes

The protocols for the rat studies were approved by the Institutional Animal Care and Use Committee at Michigan State University. Eight-week (240 g) male Long Evans rats were purchased from the Harlan laboratories (Haslett, MI, USA). Diabetes was induced by intraperitoneal injections of 65 mg STZ (Sigma-Aldrich, St. Louis, MO) per kg body wt as previously described [19]. Control animals received injections of 100 mM citric acid buffer (PH = 4.5) only. Body weight gains and blood glucose were monitored biweekly. Animals were maintained on 12 h light: 12 h dark cycle (lights on at 7∶00 am, lights off at 7∶00 pm). Circadian studies were performed 6 weeks after the induction of diabetes.

### *In vivo* Circadian Studies

Control and diabetic rats were sacrificed every 2 h beginning 1 h after the lights went on (Zeitgeber time (ZT) 1) throughout the 72 h light/dark cycle. During the dark phase the dissection was carried out under dim red light. The livers and brains were immediately excised and rinsed with ice-cold PBS to remove excess blood. The brains were kept in RNA-later (Ambion, Austin, TX, USA) until the SCNs were isolated. Brain slice, 0.8 mm in thickness, containing the SCN was made using optic chiasm as landmark [20]. A piece of hypothalamic tissue (1×0.5×0.8 mm, wide by height by depth), containing the bilateral SCN was dissected out right above the optic chiasm under a dissecting microscope using a scalpel blade. Livers were snap frozen in liquid nitrogen and stored at −80°C. To isolate retina, the eyes were enucleated, cornea, lens and vitreous humor were removed; and the retina was gently separated from choroid, washed in PBS, snap frozen in liquid nitrogen and stored at −80°C.

### Cell Culture

Primary cultures of rREC (99% pure) were prepared from the retinas isolated from 4 control rats and 4 rats with diabetes as previously described [21]. rREC were grown in six-well plates coated with 0.1% gelatin in 2 ml growth medium/well consisting of Dulbecco’s modified Eagle’s medium/F12 (1∶1 ratio, 5 mmol/l glucose) supplemented with 10% fetal bovine serum, 5% ECGS, 1% penicillin/streptomycin, and 1× ITS at 37°C in humidified 95% air and 5% CO_2_.

### Dexamethasone Exposure

rREC were grown to 80% confluence and exposed to 100 nM dexamethasone (Sigma-Aldrich, St. Louis, MO), in the absence of FBS for 2 h. After 2 h, the medium was replaced with DMEM/Ham’s F12 medium and 5% ECGS, 1% penicillin/streptomycin, and 1× ITS, supplemented with 10% FBS. The cells did not receive any further medium changes from this point until the time of harvest. Cells were harvested every 4 h up to 24 h following synchronization.

### RNA Isolation

Rat retinas were homogenized in Trizol reagent (Invitrogen, Carlsband, CA), and RNA was isolated according to manufacturer instructions. After adding chloroform, the upper aqueous phase was separated and RNA was precipitated with isopropyl alcohol, washed with 75% ethanol, and redissolved in RNase-free water.

### Quantitative Real Time-polymerase Chain Reaction (qRT-PCR)

Transcript-specific primers for each gene were designed using Primer3 software (available at http://frodo.wi.mit.edu/primer3/), and listed in Table S1. First strand cDNA was synthesized using the SuperScript II RNase H Reverse Transcriptase (Invitrogen, Carlsband, CA). Synthesized cDNA was mixed with 2x SYBR Green PCR Master Mix (Applied Biosystems) and the different sets of gene-specific forward and reverse primers, and then subjected to real-time PCR quantification using the ABI PRISM 7500 Fast Real-time PCR System (Applied Biosystems). All reactions were performed in triplicates. The relative amounts of mRNAs were calculated by using the comparative CT method. All of genes were normalized to the abundance of cyclophilin mRNA.

### Immunostaining and Quantitation of BMAL1 and SREBP1C Proteins Expression

Rat eyes were processed for standard Paraffin embedding, sectioned on a rotary microtome at 5 µm and air-dried. Sections were then deparaffinized/rehydrated through xylene and serial concentrations of ethanol. The Rodent Decloaker (Biocare Medical LIC., Concord, CA) was used for antigen unmasking and heat retrieval. After pretreatments, sections were blocked with 10% normal goat sera (Jackson ImmunoResearch Laboratories Inc., West Grove, PA) for 2 h at room temperature and incubated with polyclonal rabbit anti-BMAL 1 antibody (Abcam Inc., Cambridge, MA) at 1∶100 or polyclonal rabbit anti-SREBP 1C antibody (Santa Cruz Biotechnology, Delaware, CA) at 1∶50 in PBS with 1% BSA for 2 h at room temperature or overnight at 4°C. After washing with PBS, the FITC conjugated goat anti-rabbit IgG (1∶600) (Sigma-Aldrich, St. Louis, MO) secondary antibodies were incubated for 1 hour at room temperature in the dark. Following washing with PBS three times, nuclei were stained for 10 min with DAPI (Sigma-Aldrich, St. Louis, MO). Slides were rinsed three times with distilled water and then postfixed with the Prolong Antifade Kit (Invitrogen Life Technologies, Carlsbad, CA), covered with glass coverslips, and subjected to fluorescent microscopy. The green (FITC) fluorescence was visualized by excitation at 494 nm and collection of emissions at 518 nm, whereas the excitation and emission wavelengths for the DAPI detection were 350 and 460 nm, respectively. The images were viewed, and pictures were taken using a Nikon TE2000 fluorescence microscope equipped with Photometrics CoolSNAP HQ2 camera. All images were taken with matched exposure time for experimental and control sections. The quantitation of BMAL1 or SREBP1C protein expression was done using the MetaMorph imaging software (Molecular Devices, Downingtown, PA). The fluorescence intensity (FITC-green) of the BMAL1 or SREBP1C protein signal in the rat retina was normalized to the fluorescence intensity of DAPI in the nuclei. The autofluorescence of the section was excluded from the analysis.

### Periodicity Analysis

To identify rhythmic gene expression we used two statistical programs. First, COSOPT based on an algorithm described by *Straume M* [22] with a COSOPT multiple measures corrected β value (pMMC-Beta) cut-off of 0.05 was used. The amplitude and phase were calculated using COSOPT analysis, the data were then evaluated by single cosine R analysis to identify rhythmic gene expression. The data was considered diurnal oscillation by the zero-amplitude test with a *Pr*-value of less than 0.05.

### Statistical Analysis

Data are expressed as the mean ± SE for gene expression. Two-way ANOVA with post hoc Tukey test (GraphPad Prism5, GraphPad Software, San Diego, CA) was used to compare the data obtained from independent samples. Significance was established at p<0.05.

## Results

### Differential Daily Expression Patterns of Clock Genes in the SCN, Retina and Liver

The expression pattern of clock genes in SCN, retina and liver was examined every 2 h for the 72 h period. Expression levels of *bmal1, per1, per2* and *cry1* displayed the rhythmic oscillation pattern in SCN isolated from control and diabetic rats by R analysis (Table 1). Expression levels of *cry2* in SCN isolated from the control rats had an oscillating pattern (*Pr* value is 0.0229, R analysis), while showed a non-oscillating pattern (*Pr* value is 0.258, R analysis) in SCN from the diabetic rats (Table 1). In agreement with other studies [23], [24], [25], *CLOCK* did not exhibit rhythmic expression in SCN (*Pr* value is 0.0517 for control rats and 0.463 for diabetic rats, R analysis) (Table 1).

**Table 1:** Analysis of the expression levels and daily rhythmicity of clock genes by COSOPT and R analysis in rat SCN.

			Control *vs.* Diabetic				R analysis	
Name	Gene ID	Amplitude	Difference	*P value*	Period	Phase (ZT)	*Pr value*	Daily rhythmicity
*CLOCK*	Control		no	0.118			0.0517	no
	Diabetic						0.463	no
*bmal1*	Control	8.58E-03	yes	0.002**	24	3	0.00383**	**yes**
	Diabetic	1.13E-02			24.3	1	<0.0001***	**yes**
*per1*	Control	2.33E-02	no	0.4815	21.5	7	0.0228*	**yes**
	Diabetic	2.70E-02			20.2	5	0.0408*	**yes**
*per2*	Control	4.52E-02	no	0.4239	23.4	9	0.00344**	**yes**
	Diabetic	3.40E-02			21.3	7	0.0112*	**yes**
*cry1*	Control	2.38E-02	no	0.9297	24.9	7	0.0291*	**yes**
	Diabetic	2.35E-02			26.3	9	<0.0001***	**yes**
*cry2*	Control	5.77E-03	yes	0.0473*	23.6	7	0.0229*	**yes**
	Diabetic						0.258	no

Amplitude, mean amplitude of the identified time series (theoretically zero); Period, mean period of the identified time series (theoretically 24); Phase, mean time of acrophase of the identified time series (theoretically zero); *Pr* values have been calculated by the single cosine R analysis. Genes with *Pr* value of <0.05 were considered circadianly regulated. *p<0.05, **p<0.01, ***p<0.0001.

bmal1: brain and muscle aryl-hydrocarbon receptor nuclear translocator-like 1; CLOCK: Circadian locomotor output cycles kaput; cry: cryptochrome; per: period.

**Figure 1:**
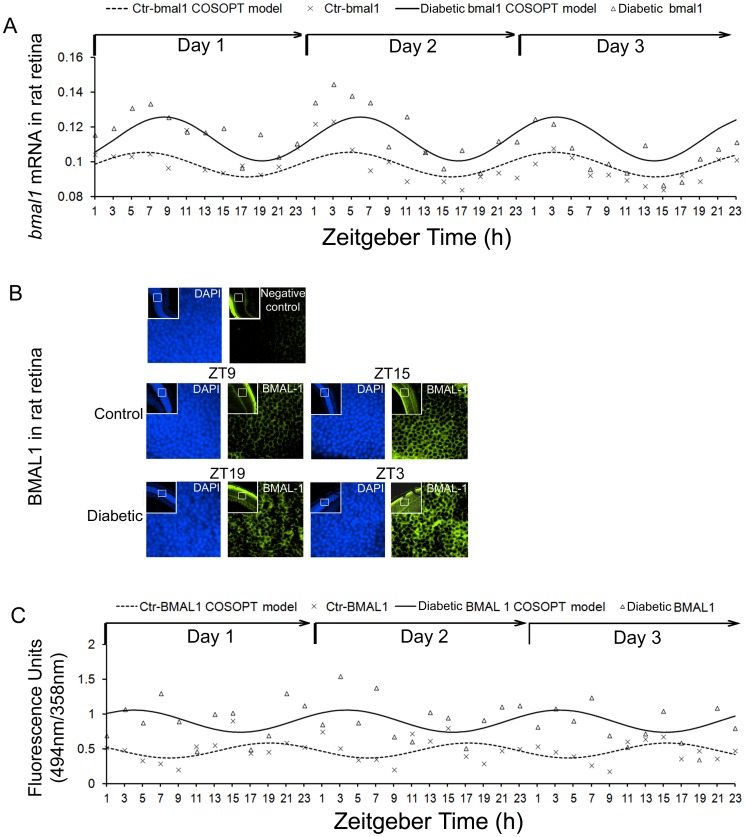
Expression profiles of *bmal1* in rat retinas. Retinas were collected every 2/dark cycles from STZ-induced diabetic rats and age matched control rats. (A) The mRNA expression of *bmal1* in retina was measured by real-time PCR and normalized to cyclophilin. COSOPT analysis was performed to analyze the rhythmic mRNA expression pattern of *bmal1*. (B) Representative diagram showed the immunoreactivity of BMAL1in the retina. Signal detection and image acquisition were performed in fluorescence microscope. (C) The fluorescence intensity (FITC-green) of BMAL1 in the retina was quantified per time point in triplicate using the MetaMorph imaging software, and the fluorescence intensity of the BMAL1 protein signal was normalized to DAPI in the nuclei. COSOPT and R analysis were performed to analyze the rhythmic protein expression pattern of BMAL1.

Moreover, periodicity analysis by COSOPT demonstrated that expression levels of all examined clock genes (*CLOCK*, *bmal1*, *per1*, *per2*, *cry1* and *cry2*) in livers isolated from both control and diabetic rats had a rhythmic oscillation pattern (Table 2). In the retina, only *bmal1*, *per1* and *cry1* exhibited the rhythmic oscillation pattern expression in both of control and diabetic rats by COSOPT or R analysis (Table 2) (Figure 1A). Interestingly, expression levels of *CLOCK* in retina isolated from the control rats had an oscillating pattern (*Pr* value is 0.00151, R analysis), while showed a non-oscillating pattern (*Pr* value is 0.692, R analysis) in retina from the diabetic rats (Table 2).

**Table 2:** Analysis of the expression levels and daily rhythmicity of clock genes by COSOPT or R analysis in rat retina and liver.

				Control *vs.* Diabetic					
Name	Tissue	Gene ID	Amplitude	Difference	*P value*	Period	Phase (ZT)	pMMC -Beta/*Pr value*	Circadianly rhythmic
*CLOCK*	retina	Control	2.13E-02	yes	0.0062**	21.6	11	***^Pr^*** 0.00151**	**yes**
		Diabetic						*^Pr^*0.692	no
	liver	Control	3.61E-03	no	0.9732	26.3	1	0.00006***	**yes**
		Diabetic	3.57E-03			21.4	5	0.00062**	**yes**
*bmal1*	retina	Control	9.15E-02	yes	0.004**	22.3	7	0.04508*	**yes**
		Diabetic	1.21E-01			21.4	9	0.00741**	**yes**
	liver	Control	1.10E-02	no	0.2441	24.4	1	0.00001***	**yes**
		Diabetic	6.16E-03			23.2	3	0.00003***	**yes**
*per1*	retina	Control	1.87E-02	yes	0.0139*	23.8	9	***^Pr^*** 0.0166*	**yes**
		Diabetic	3.56E-03			24.1	7	***^Pr^*** 0.021*	**yes**
	liver	Control	4.22E-03	no	0.2614	23.6	7	0.00695**	**yes**
		Diabetic	6.03E-03			23.8	9	0.00465**	**yes**
*per2*	retina	Control		no	0.4719			0.89791	no
		Diabetic						0.41809	no
	liver	Control	9.98E-03	yes	0.0146*	24.2	5	0.00003***	**yes**
		Diabetic	4.23E-03			23.4	7	0.00012**	**yes**
*Cry1*	retina	Control	1.03E-01	no	0.0725	21.2	9	***^Pr^*** 0.0478*	**yes**
		Diabetic	1.09E-01			20.9	7	***^Pr^*** 0.00081**	**yes**
	liver	Control	2.41E-02	no	0.6727	24.3	3	0.00002***	**yes**
		Diabetic	2.11E-02			23.1	3	0.00001***	**yes**
*Cry2*	retina	Control		no	0.3403			0.81743	no
		Diabetic						0.74983	no
	liver	Control	4.28E-03	yes	0.0053**	25	7	0.011083*	**yes**
		Diabetic	3.12E-03			24.7	11	0.01344*	**yes**

Amplitude, mean amplitude of the identified time series (theoretically zero); Period, mean period of the identified time series (theoretically 24); Phase, mean time of acrophase of the identified time series (theoretically zero); pMMC-Beta, mean multiple measures corrected significance probability β value. Genes with a period of between 20 and 28 hr with pMMC-β value of <0.05 were considered circadianly regulated. *Pr* values have been calculated by the single cosine R analysis. Genes with *Pr* value of <0.05 were considered circadianly regulated. *p<0.05, **p<0.01, ***p<0.0001.

### Diabetes-induced Alterations in the mRNA Expression Rhythms of Clock Genes in the SCN, Retina and Liver

To examine whether the oscillatory patterns of gene expression in SCN, retina and liver are affected by diabetes, we compared the expression rhythms of transcripts encoding positive (*CLOCK*, *bmal1*) and negative (*per1*, *per2*, *cry1* and *cry2)* arm genes in the SCN, retina and liver from control and 6-week diabetic rats. As shown in Table 1 and Table 2, diabetic animals had higher amplitude of expression of the positive arm gene *CLOCK* (p = 0.0062) in the retina but not SCN and liver; expression of *bmal1* had higher amplitude in the SCN (p = 0.002) and retina (p = 0.004) of diabetic animals, but not liver (p = 0.2441, COSOPT analysis) (Table 1, 2). The circadian pattern of *bmal1* was phase-advanced by 2 h in diabetic retina (Fig. 1A). In agreement with the mRNA expression data, the immunofluorescence intensity of BMAL 1 displayed the rhythmic oscillation pattern in both of control and diabetic rats (*Pr* value is 0.02307 for control rats and 0.0108 for diabetic rats, R analysis) (Table 3) and was significantly upregulated by diabetes in the retinas (p = 0.002, COSOPT analysis) (Table 3) (Figure 1B, C).

**Table 3:** Analysis of fluorescence intensity and daily rhythmicity of BMAL1 and SREBP1C by COSOPT and R analysis in rat retina.

			Control *vs.* Diabetic				R analysis	
Name	Gene ID	Amplitude	Difference	*P value*	Period	Phase (ZT)	*Pr value*	Daily rhythmicity
BMAL1	Control	4.74E-01	yes	0.002**	22.2	3	0.02307*	**yes**
	Diabetic	1.16E+00			23.7	3	0.0108*	**yes**
SREBP 1C	Control	2.86E-01	yes	0.0007***	24.8	3	0.0497*	**yes**
	Diabetic	6.74E-01			23.5	11	<0.0001***	**yes**

Amplitude, mean amplitude of the identified time series (theoretically zero); Period, mean period of the identified time series (theoretically 24); Phase, mean time of acrophase of the identified time series (theoretically zero); *Pr* values have been calculated by the single cosine R analysis. Genes with *Pr* value of <0.05 were considered circadianly regulated. *p<0.05, **p<0.01, ***p<0.0001.

Diabetes had a strong inhibitory effect on the negative arm genes. As shown in Tables 1 and 2, *per1* had lower amplitude and was phase shifted in the retina (p = 0.0139); *cry2* had lower amplitude in SCN (p = 0.0473) and liver (p = 0.0053), *Per2* had lower amplitude and was phase shifted in the liver (p = 0.0146).

### Effect of Diabetes on Daily Expression of the Genes Controlling Lipid Metabolism in the Retina

We next determined whether the expression profile of the key lipid metabolic regulator, *PPARα* and *PPARγ*, exhibited rhythmicity in the retina and liver, and whether this rhythm was impaired in diabetes. Periodicity analysis demonstrated that expression levels of *PPARα* in retina and liver isolated from both of control and diabetic rats had a rhythmic oscillation pattern (pMMC-β <0.05, COSOPT analysis), but amplitude of *PPARα* expression didn’t show any change between control and diabetic rats (p>0.05, COSOPT analysis) (Table 4). Expression levels of *PPARγ* in retina isolated from control and diabetic rats had a rhythmic oscillating pattern, while displayed the non-oscillation pattern expression in liver (pMMC-β <0.05, COSOPT analysis) (Table 4). Interestingly, diabetic rats had a lower amplitude of *PPARγ* in liver compared with control rats (p = 0.0255, COSOPT analysis) (Table 4).

**Table 4:** Analysis of the expression levels and daily rhythmicity of lipid metabolism related-genes by COSOPT or R analysis in rat retina and liver.

				Control *vs.* Diabetic					
Name	Tissue	Gene ID	Amplitude	Difference	*P value*	Period	Phase (ZT)	pMMC -Beta/*Pr value*	Circadianly rhythmic
*PPARα*	retina	Control	2.53E-02	no	0.3636	21.9	7	0.01191*	**yes**
		Diabetic	2.87E-02			24	9	0.00749**	**yes**
	liver	Control	9.51E-03	no	0.8354	22.7	7	0.004**	**yes**
		Diabetic	1.01E-02			23.1	7	0.0001***	**yes**
*PPARγ*	retina	Control	2.99E-04	no	0.9536	27.5	5	0.04038*	**yes**
		Diabetic	3.01E-04			23.6	11	0.01457*	**yes**
	liver	Control		yes	0.0255*			0.2956	no
		Diabetic						0.9189	no
*srebp1c*	retina	Control		yes	0.0071**			0.96673	no
		Diabetic						0.21764	no
	liver	Control	1.01E-01	yes	0.0003***	26.5	3	0.0358*	**yes**
		Diabetic	1.06E-02			27.7	5	0.008**	**yes**
*Elovl2*	retina	Control	1.45E-02	yes	0.001***	22.3	9	***^Pr^*** 0.0015**	**yes**
		Diabetic	1.16E-02			21.7	7	***^Pr^*** 0.0452*	**yes**
	liver	Control		no	0.1197			0.3311	no
		Diabetic						0.322	no
*Elovl4*	retina	Control	5.12E-01	no	0.4075	21.4	9	***^Pr^*** 0.0368*	**yes**
		Diabetic						*^Pr^* 0.7429	no

Amplitude, mean amplitude of the identified time series (theoretically zero); Period, mean period of the identified time series (theoretically 24); Phase, mean time of acrophase of the identified time series (theoretically zero); pMMC-Beta, mean multiple measures corrected significance probability β value. Genes with a period of between 20 and 28 hr with pMMC-β value of <0.05 were considered circadianly regulated. *Pr* values have been calculated by the single cosine R analysis. Genes with *Pr* value of <0.05 were considered circadianly regulated. *p<0.05, **p<0.01, ***p<0.0001.

**Figure 2:**
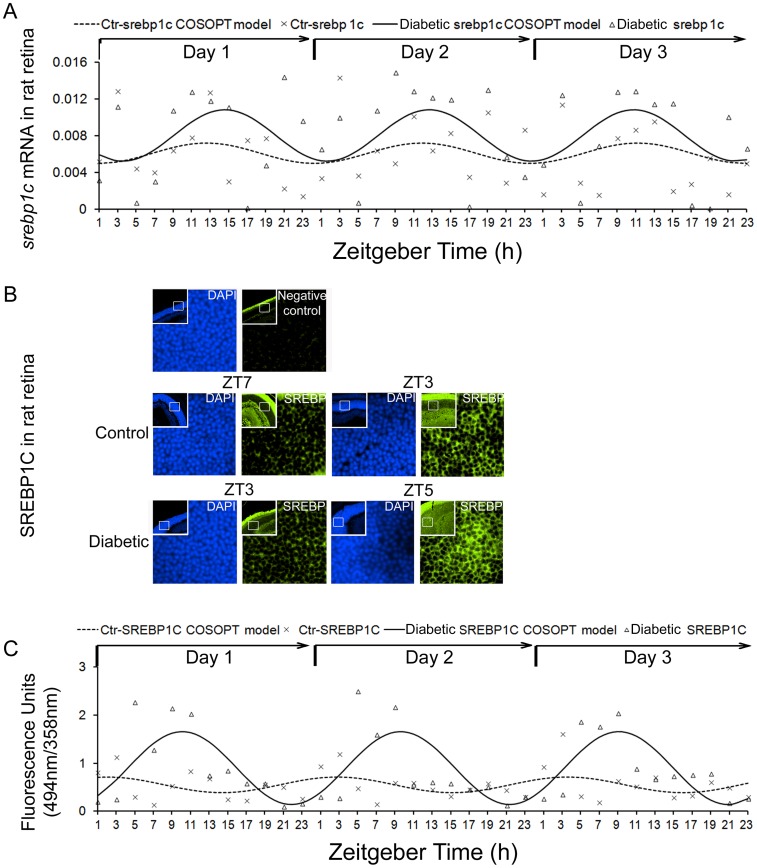
Expression profiles of *srebp1c* in rat retinas. Retinas were collected every 2/dark cycles from STZ-induced diabetic rats and age matched control rats. (A) The mRNA expression of *srebp1c* in retina was measured by real-time PCR and normalized to cyclophilin. COSOPT analysis was performed to analyze the rhythmic mRNA expression pattern of *srebp1c*. (B) Representative diagram showed the immunoreactivity of SREBP1C in the retina. Signal detection and image acquisition were performed in fluorescence microscope. (C) The fluorescence intensity (FITC-green) of SREBP1C in the retina was quantified per time point in triplicate using the MetaMorph imaging software, and the fluorescence intensity of the SREBP1C protein signal was normalized to DAPI in the nuclei. COSOPT and R analysis were performed to analyze the rhythmic protein expression pattern of SREBP1C.

It has been reported that *srebp1c* exhibits a 24-hour pattern in the liver [26]. We next examined *srebp1c* expression profile in control and diabetic retina and liver. As shown in Table 4, *srebp1c* mRNA expression exhibited rhythmic oscillation pattern expression in the liver but not retina (pMMC-β <0.05, COSOPT analysis), which was consistent with the previous report [26]. Notably, diabetic animals had higher amplitude of *srebp1c* (p = 0.0071, COSOPT analysis) (Table 4, Figure 2A) in retina, lower amplitude in the liver (p = 0.0003, COSOPT analysis) (Table 4). Despite the lack of rhythmic oscillation pattern of mRNA, SREBP1C protein was rhythmically expressed (*Pr* value is 0.0497 for control rats and <0.0001 for diabetic rats, R analysis) and had higher amplitude in diabetic retinas (p = 0.0007, COSOPT analysis) (Table 3) (Figure 2B, C).

Our previous data has identified that Elovl4 had the highest expression level among all the fatty acid elongases in the retina and was not expressed in the liver [19]. Retinas also had high levels of Elovl2 expression [19]. *Elovl2* expression showed a rhythmic oscillation pattern in the retina (*Pr* value is 0.0015 for control rats and 0.0452 for diabetic rats, R analysis) but not in the liver (Table 4). Interestingly, expression level of *Elovl2* was significantly decreased in the retina from diabetic rats compared with control rats (p = 0.001, COSOPT analysis) but was not changed in the liver (Table 4). Expression level of *Elovl4* exhibited rhythmic oscillation pattern in the retina from control rats (*Pr* value is 0.0368, R analysis) but not diabetic rats (Table 4).

### Diabetes Induced an Increase of Circadian Oscillator Gene Expression in rREC

**Figure 3:**
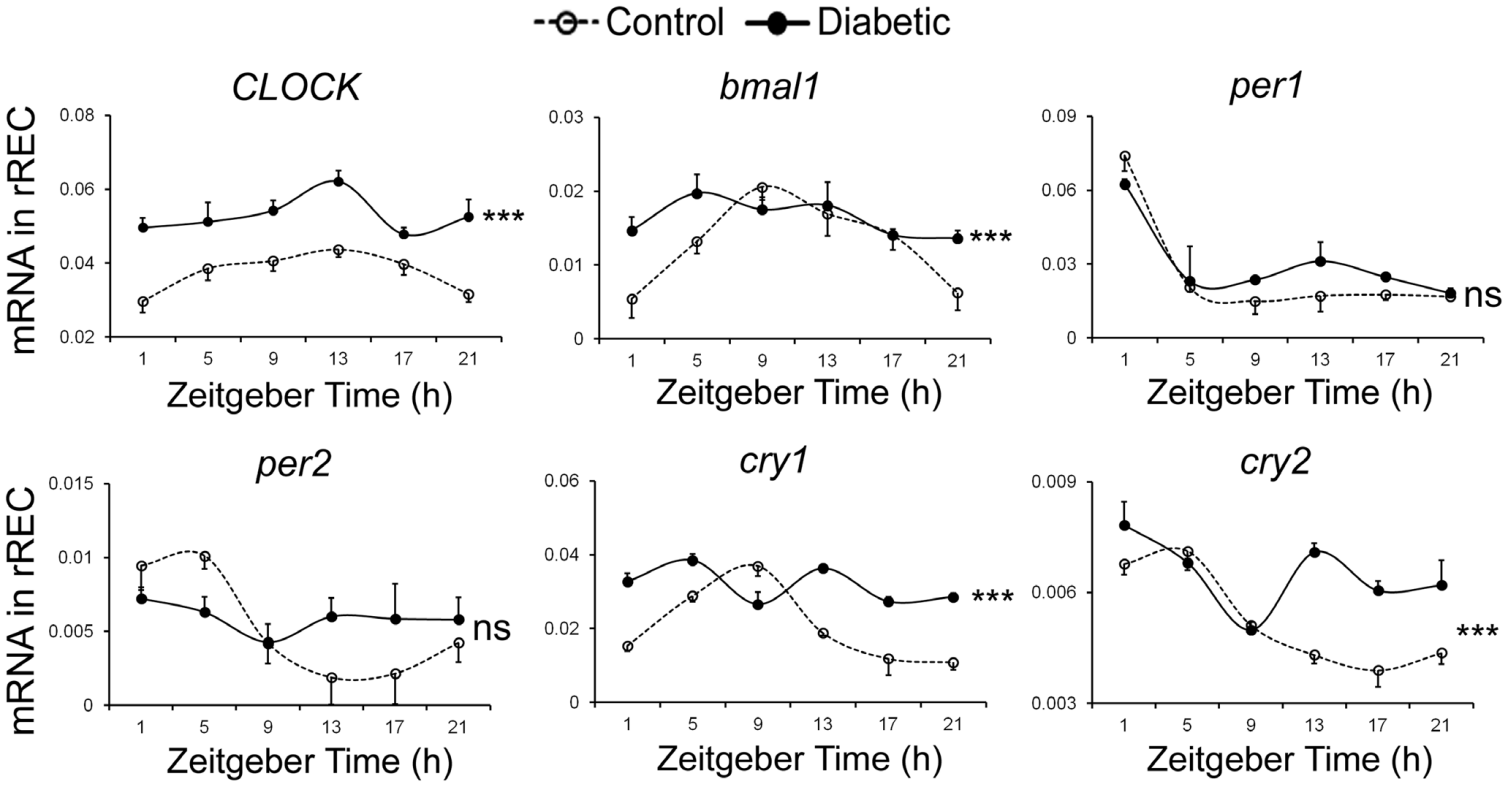
The expression profiles of core circadian oscillators in synchronized rREC. Cultures of rREC isolated from control and diabetic donors were exposed to 100-time PCR analysis was performed to examine the expression and level of *CLOCK*, *bmal1*, *per1*, *per2*, *cry1* and *cry2*. Expression values are normalized to cyclophilin. Values are shown as the mean ± SE, n = 3 for observations in rREC from control and diabetic rats. Statistical analysis was performed using two-way ANOVA for diabetes and time effect, and Dunnett’s post-test to compare replicates by time point, *p<0.05, **p<0.01,***p<0.001, ns means not significant.

Retinal vasculature is the primary target tissue affected by DR, however it comprises very small percentage of retinal tissues, thus changes occurring in the vasculature could be lost in the whole retina analysis. To model the effect of diabetes on the circadian rhythm and level of clock genes expression in the vasculature, we used primary rREC isolated from control and diabetic rats. Expression levels of *CLOCK*, *bmal1*, *cry1* and *cry2* were significantly increased in rREC from diabetic rats compared with control (p<0.0001, two-way ANOVA) (Figure 3).

### Expression Profiles of Lipid Metabolism-related Genes in the rREC

**Figure 4:**
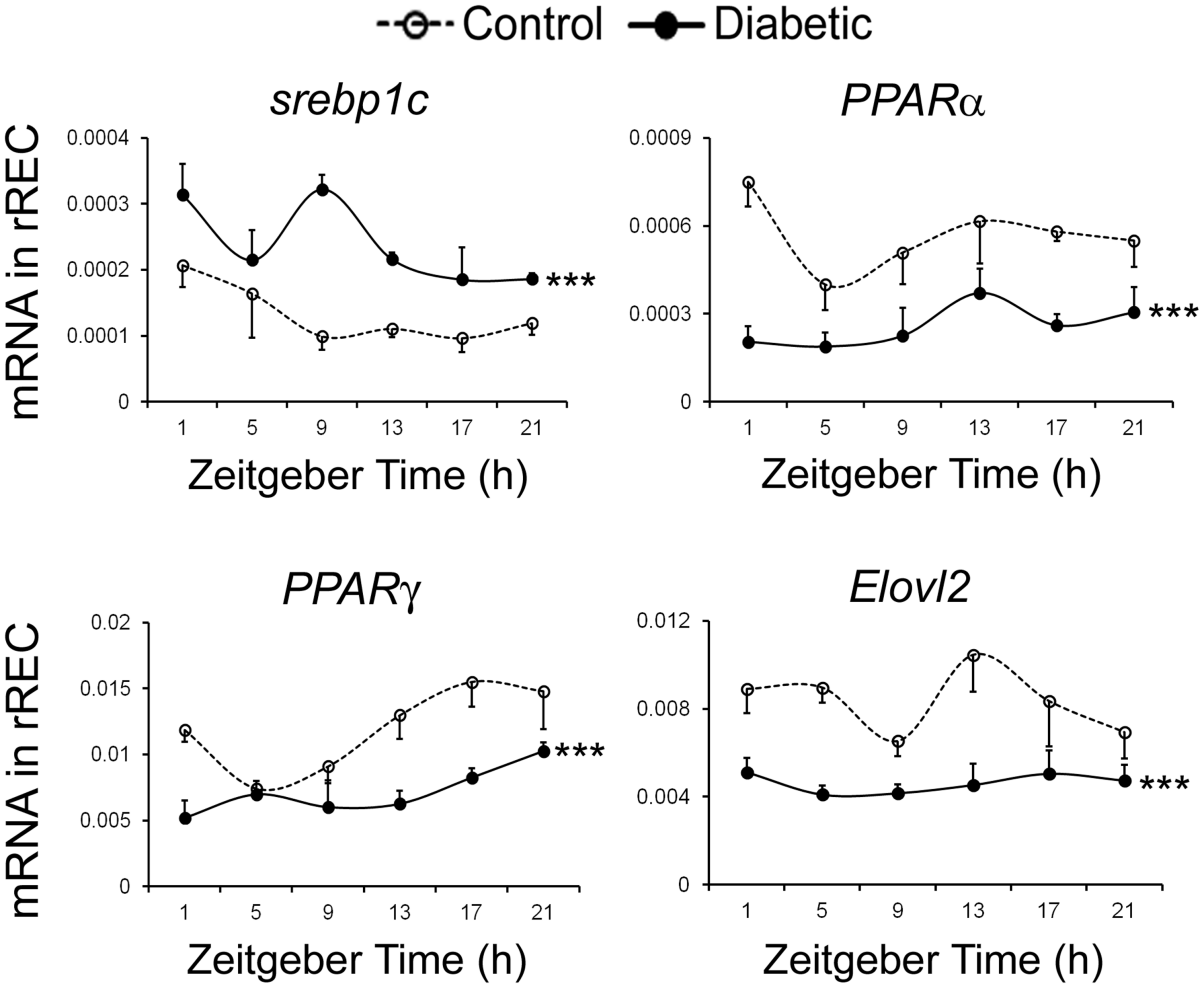
The expression profiles of lipid metabolism-related genes in synchronized rREC. Cultures of rREC isolated from control and diabetic donors were exposed to 100*PPARα*, *PPARγ, srebp 1c, Elovl2* and *Elovl4*. Expression values are normalized to cyclophilin. Values are shown as the mean ± SE, n = 3 for observations in rREC from control and diabetic rats. Statistical analysis was performed using two-way ANOVA for diabetes and time effect, and Dunnett’s post-test to compare replicates by time point, *p<0.05, **p<0.01,***p<0.001, ns means not significant.

Next, expression levels of *srebp1c*, *PPARα*, *PPARγ, Elovl2* and *Elovl4* mRNA were analyzed in the cultured rREC from control and diabetic rats for up to 24 h following synchronization. As shown in Figure 4, rREC isolated from diabetic rats had higher expression of *srebp1c*; lower expression of *PPARα, PPARγ* and *Elovl2* compared with control (p<0.0001, two-way ANOVA) (Figure 4), which was consistent with the in vivo data (Figure 3). Notably, *Elovl4* had very low expression level in rREC.

## Discussion

In mammals circadian timing system includes not only the central master circadian pacemaker located in the SCN but also peripheral oscillators distributed throughout the body [2]. Circadian system plays an important role in regulating physiology and behavior. Circadian control is entrained by external signals such as light, food intake, temperature and social interactions. Central circadian clock in SCN is mainly synchronized by light and temperature sensed by the retina. In this way, signals from daily light–dark and temperature oscillations cycle reach SCN from the retina, and then SCN generates output signals that reach the rest of the peripheral tissues. Several studies have strongly supported the notion that mammalian retina contains an intrinsic circadian clock [27], [28], which is able to regulate a number of cellular, biochemical, and physiological processes. The retinal clock can generate circadian rhythmicity independent of the master circadian clock located in SCN [29]. Global transcriptomic gene expression profiles have shown that an estimated 9% of the genes in SCN, heart, and liver exhibited circadian expression profile [5], [6], [30], [31], however, only 8–10% of these circadian genes shared similar pattern between SCN and individual peripheral tissues [5], supporting tissue specific circadian expression profiles for majority of genes.

In this study expression of *bmal1* showed rhythmic oscillation pattern in the SCN, retina and liver of rats, supporting the notion that intracellular circadian clock system operates not only in SCN but also in peripheral tissues and that peripheral oscillators are not directly controlled by SCN. Consistent with our results, it has been reported that *CLOCK* expression is constitutive in the SCN, but cyclic in the peripheral tissues [32]. Moreover, our data demonstrate distinct circadian expression patterns of *per1, per2*, *cry1* and *cry2* among different tissues.

The role of clock genes in the retinal health was previously demonstrated by others and us [33], [34]. We demonstrated that microvascular repair is a highly synchronized process requiring proper function on both central and peripheral circadian system [34]. Diabetes-induced disruption of daily expression patterns of clock genes and clock-controlled genes could lead to the loss of synchronization of retinal repair cues and release and migration of endothelial progenitor cells required for the efficient repair process. Hyperglycemia-induced chronic inflammation and tissues damage coupled with deficient repair process due to the lack of synchronization would lead to exacerbated microvascular damage and diabetic retinopathy [35]. Indeed, we have previously demonstrated that *per2* knock-out animals develop diabetes-like retinopathy [33].

Although it is now well accepted that diabetes alters profiles of rhythmic clock gene expression both at the central and peripheral levels, the mechanism of this effect, the exact clock genes involved and the nature of changes in clock genes are still largely unknown. Hyperglycemia was shown to decrease the expression level of the negative clock arm genes, *per 1* and *per 2* in cultured Rat-1 fibroblasts [35], *per1* and *per2* gene expression was downregulated in the liver [36], [37] and kidney [38] of diabetic mice, and the degree of downregulation was dependent on the severity of diabetes [39]. Moreover, expression levels of *per2* and *cry2* were significantly lower in islets from human donors with type 2 diabetes [40] and mRNA expression levels of *per1* and *per2* were significantly lower in the leucocytes of patients with type 2 diabetes than in non-diabetic controls [41]. The present study further establishes the inhibitory effect of diabetes on the negative clock arm. We demonstrated that the expression level and the amplitude of rhythmic oscillations of the *per1* were dramatically decreased in the retina, *per2* was decreased in the liver and *cry2* lost circadian pattern in the SCN on diabetic rats.

Downregulation of the negative clock arm in diabetes is often, but not always [42], accompanied by higher amplitude of the positive clock arm, *CLOCK* and *bmal1*. *Bmal1* was increased in the pineal gland of STZ-treated Wistar rats [43]. In this study we found an increase in *bmal1* amplitude in the SCN; an increase in *CLOCK* and *bmal1* amplitude, and two hour phase advance in *bmal1* rhythmic oscillations in the retinas of diabetic rats. Interestingly, the effect of diabetes on the expression of clock genes in the retina was more pronounced than in the SCN or liver.

Recent studies have indicated that disruption of circadian clock is sufficient to affect glucose and lipid metabolism [44], [45]. Metabolic dysfunction, in turn, reportedly induces dysregulation of circadian rhythms in the periphery. SREBP1C plays a central role in transcriptional regulation of genes controlling lipid metabolism, and *srebp1* expression in the liver shows circadian rhythm [46]. In agreement with previous study, our data also showed that *srebp1c* had rhythmic oscillation expression pattern in the liver. Notably, we report that the mRNA expression of *srebp1c* exhibits rhythmic oscillation pattern in the retina and both mRNA and protein rhythmic expression is modified by diabetes. Our data also demonstrated that SREBP1C protein oscillations do not follow the pattern of mRNA oscillations. These results reflect complex SREBP translational and post-translational regulation. SREBP1C binds and activates its own *srebp1c* promoter [47], [48], [49], [50]. Moreover, it is well-known that protein levels of SREBPs are regulated by proteolytic maturation and ubiquitination-dependent degradation in response to nutritional and hormonal changes [47].

PPARα is another key regulator gene expression controlling cellular metabolism implicated in the development of diabetic retinopathy. *PPARα* gene is a circadian clock-regulated gene in the liver [51], [52]. It has been shown by chromatin immunoprecipitation that PPARα directly binds to the *bmal1* promoter [53]. Our data demonstrate that there is rhythmic oscillation pattern of *PPARα* mRNA expression in retina and liver.

Recent studies suggest that PPARγ plays an important role in regulating the molecular circadian clocks and metabolic pathways, and could directly regulate the transcriptional level of *bmal1* [54], [55]. *PPARγ* expression, however, exhibited no circadian rhythm in the livers of STZ induced diabetic or spontaneous type 1 diabetic rats [42]. In agreement with these results, our data also showed that *PPARγ* expression was non-rhythmic in the liver of STZ-induced diabetic rat. Furthermore, we found that the expression level of *PPARγ* was downregulated in the retina and liver from STZ-induced diabetic rats, which was in accordance to other studies [56], [57].

We previously showed that fatty acid elongases *Elovl2* and *Elovl4* are reduced in diabetic retinopathy [19], thus, we examined their daily expression in the retina and liver. Both of *Elovl2 and Elovl4* had a rhythmic oscillation pattern in the control retina but not in the liver. Diabetic retinas lost *Elovl4* rhythmic oscillation and had lower amplitude of *Elovl2* oscillations. Taken together, these results demonstrate that genes centrally involved in the regulation of lipid metabolism, *srebp1c*, *PPARγ* and *PPARα*, *Elovl4* and *Elovl2* have daily rhythms of expression profile in the retina and their daily rhythms are perturbed by diabetes.

Daily expression rhythms in the whole retina provide us with valuable information on overall changes; however it is mainly contributed by the neuroretina. To determine if the primary target tissue affected by DR, retinal vasculature, is affected in a similar way, we used primary rREC isolated from control and diabetic rats. Several cell lines and primary cell cultures isolated from peripheral organs were recently shown to exhibit circadian rhythms after synchronization by glucocorticoid or high concentrations of serum [58], [59], [60], [61]. In our study synchronized rREC derived from diabetic rats had higher expression level of circadian clock genes (*CLOCK*, *bmal1*, *cry1* and *cry2*) and *srebp1c*; lower expression level of *PPARα, PPARγ* and *Elovl2* compared to rREC isolated from control rats. These data demonstrate that regulation of circadian expression of clock genes and lipid metabolism-related genes is similarly affected by diabetes in retinal vasculature as well as in the whole retina.

The main conclusion of this study is that diabetes differentially disrupts circadian regulation of core clock genes and lipid metabolism-related genes in SCN, retina and liver with most significant changes occurring in the retina. One of the major consequences of the disruption of circadian clock is altered circadian expression patterns of genes regulating lipid metabolism in the retina. Retina is a highly cyclic tissue with metabolic demands, inflammatory processes and vascular repair varying greatly during the light and dark cycles. Loss of synchronization between retinal metabolic demands and lipid metabolism in diabetes could contribute to the pathogenesis of diabetic retinopathy.

## Supporting Information

Table S1 **Rat primer pairs used for qPCR.** The gene accession numbers and sequences were used for primer design.
(DOCX)
