# Supplementary material for: Changes in the Daily Rhythm of Lipid Metabolism in the Diabetic Retina
Source: PLoS One. 2014 Apr 15;9(4):e95028. doi: 10.1371/journal.pone.0095028 (PMC3988159; doi:10.1371/journal.pone.0095028)
Supplement: Table S1 — Rat primer pairs used for qPCR. The gene accession numbers and sequences were used for primer design. (DOCX) [file pone.0095028.s001.docx]

Table S1. Rat primer pairs used for qPCR

| Target Gene | GeneBank Accession Number | Primer Sequence | |
| --- | --- | --- | --- |
| CYCLOPHILIN A | NM_017101 | | (F) 5’-CTTCTTGCTGGTCTTGCCATTCCT-3’  (R) 5’-TGGATGGCAAGCATGTGGTCTTTG-3’ |
| SREBP-1C | AF286470 | | (F) 5’-CATGGATTGCACATTTGAAGAC-3’  (R) 5’-GCAGGAGAAGAGAAGCTCTCAG-3’ |
| PPAR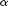 | NM_013196 | | (F) 5’-ACTAGCAACAATCCGCCTTTTG-3’  (R) 5’-GGACCTCTGCCTCCTTGTTTTC-3’ |
| PPARγ | NM_013124 | | (F) 5’- GGAAAAAACCCTTGCATCCTTC -3’  (R) 5’- TTCAAACTCCCTCATGGCCA -3’ |
| ELOVL4 | NM_001191796 | | (F) 5’-GAAGTGGATGAAAGACCGAGA -3’  (R) 5’-GCGTTGTATGATCCCATGAA-3’ |
| ELOVL2 | NM_001109118 | | (F) 5’-TTTGGCTGTCTCATCTTCCA-3’  (R) 5’-GGGAAACCATTCTTCACTTC-3’ |
| CLOCK | NM_021856 | | (F) 5’-CAGCAGTCTCAGACCCTTCC -3’  (R) 5’-ACCTCCGCTGTGTCATCTTC -3’ |
| BMAL 1 | NM_024362 | | (F) 5’-AAGTGCAACAGGCCTTCAGT -3’  (R) 5’-CAAGTAGCCTGTGCTGTGGA -3’ |
| CRY 1 | NM_198750 | | (F) 5’-TCAGTTGGGAAGAAGGGATG -3’  (R) 5’-AGGGCAGTAGCAGTGGAAAA -3’ |
| CRY2 | NM_133405 | | (F) 5’-CAGGTATCGCCGGATGTAGT-3’  (R) 5’-GTCCTGCAGTGCTTTCTTCC-3’ |
| PER1 | NM_001034125 | | (F) 5’-AGCAAATCAACTGCCTGGAC -3’  (R) 5’-AGGAGGCACATTTACGCTTG -3’ |
| PER2 | NM_031678 | | (F) 5’-CAGTGGCAAGAGTCAAAGCA -3’  (R) 5’-TTGCATTTCTGCAACAGAGG -3’ |
